# Worry is associated with robust reductions in heart rate variability: a transdiagnostic study of anxiety psychopathology

**DOI:** 10.1186/s40359-016-0138-z

**Published:** 2016-06-03

**Authors:** John A. Chalmers, James A. J. Heathers, Maree J. Abbott, Andrew H. Kemp, Daniel S. Quintana

**Affiliations:** School of Psychology, University of Sydney, Sydney, Australia; Division of Cardiology, Poznań University of Medical Sciences, Poznań, Poland; Discipline of Psychiatry, University of Sydney, Sydney, Australia; Department of Psychology, Swansea University, Swansea, UK; NORMENT, KG Jebsen Centre for Psychosis Research, Division of Mental Health and Addiction, University of Oslo, and Oslo University Hospital, Oslo, Norway; NORMENT, KG Jebsen Centre for Psychosis Research, Building 49, Oslo University Hospital, Ullevål, Kirkeveien 166, PO Box 4956, Nydalen N- 0424 Oslo, Norway

**Keywords:** Psychophysiology, Autonomic nervous system, ANS, Heart rate variability, HRV, Anxiety, Worry, Dimensional-trait models

## Abstract

**Background:**

Individuals with anxiety disorders display reduced resting-state heart rate variability (HRV), although findings have been contradictory and the role of specific symptoms has been less clear. It is possible that HRV reductions may transcend diagnostic categories, consistent with dimensional-trait models of psychopathology. Here we investigated whether anxiety disorders or symptoms of anxiety, stress, worry and depression are more strongly associated with resting-state HRV.

**Methods:**

Resting-state HRV was calculated in participants with clinical anxiety (*n* = 25) and healthy controls (*n* = 58). Symptom severity measures of worry, anxiety, stress, and depression were also collected from participants, regardless of diagnosis.

**Results:**

Participants who fulfilled DSM-IV criteria for an anxiety disorder displayed diminished HRV, a difference at trend level significance (*p* = .1, Hedges’ *g* = -.37, BF_10_ = .84). High worriers (Total *n* = 41; *n* = 22 diagnosed with an anxiety disorder and *n* = 19 not meeting criteria for any psychopathology) displayed a robust reduction in resting state HRV relative to low worriers (*p* = .001, Hedges’ *g* = -.75, BF_10_ = 28.16).

**Conclusions:**

The specific symptom of worry – not the diagnosis of an anxiety disorder – was associated with the most robust reductions in HRV, indicating that HRV may provide a transdiagnostic biomarker of worry. These results enhance understanding of the relationship between the cardiac autonomic nervous system and anxiety psychopathology, providing support for dimensional-trait models consistent with the Research Domain Criteria framework.

**Electronic supplementary material:**

The online version of this article (doi:10.1186/s40359-016-0138-z) contains supplementary material, which is available to authorized users.

## Background

Anxiety disorders are the most prevalent of the psychiatric disorders [1], and the most costly [2]. Anxiety disorders carry a three to four-fold increased risk of cardiovascular disease (CVD) after accounting for gender, substance use, and depression, [3–5] and a two-fold increased risk for cardiac mortality [6–8]. Reductions in resting-state heart rate variability (HRV) reflect cardiac autonomic dysfunction, which plays a key role in the development of cardiovascular diseases. Although reductions in HRV may provide a link between anxiety and ill health [9–13], past studies on anxiety disorders have reported contradictory findings. Here we sought to determine whether anxiety disorders or their symptoms spanning a non-clinical to clinical spectrum are associated with stronger relations with HRV.

HRV indexes the complex modification of heart rate over time and has become a widely used measure of autonomic control of heart rate. Low HRV is associated with a wide variety of psychological states, behaviours and conditions including reduced capacity for self-regulation, enhanced withdrawal behaviours, psychiatric illness and cardiovascular disease [14–19] leading us to suggest previously that HRV may help to elucidate the pathways linking mental and physical health [13]. Anxiety disorders have been characterised by low HRV [20] and two complementary models – polyvagal theory and neurovisceral integration – provide a platform on which these findings may be interpreted.

Polyvagal theory [21] links high resting-state HRV to social engagement and effective emotion regulation strategies, while low HRV is linked to withdrawal behaviours [21], a characteristic that may underpin many of the anxiety disorders. An alternative biobehavioural theory, the neurovisceral integration model [22, 23], further underscores the important inhibitory role of vagal activity in emotion regulation. This model outlines specific central and peripheral pathways that connect autonomic, attentional, and affective systems involved in emotion regulation. The model suggests that the integrity of these pathways may be compromised in anxiety disorders, such that the central and autonomic nervous systems are rigidly coupled, resulting in difficulty with disengaging from and inhibiting threat detection (e.g., hyper-vigilance, apprehension, avoidance, panic sensations, increases in heart rate, and decreases in HRV).

While studies on HRV in the anxiety disorders have reported contradictory findings, recent meta-analytic work has established that anxiety disorders are associated with poor autonomic function [24–26]. However, it remains unclear whether specific symptoms characteristic to the anxiety disorders or the disorder itself are characterised by the most robust associations. The finding that reduced HRV is a common feature of anxiety disorders (except perhaps obsessive-compulsive disorder; OCD) [20] may be interpreted within a dimensional-trait model of psychopathology [27] in which HRV reductions may reflect a failure to inhibit stereotypical fight-flight-freeze behavioural responses [21–23]. However, prior studies have typically focused on distinct nosological disorders rather than on transdiagnostic features of anxiety symptomatology. This is an important distinction because most psychological disorders are heterogeneous and symptoms may also be present in individuals that do not meet formal diagnostic criteria. Moreover, anxiety symptomatology is present in a wide range of psychiatric disorders. Therefore, in addition to comparing HRV in those with and without an anxiety disorder, we also determined whether participants high versus low on specific symptoms are associated with more robust reductions in HRV, regardless of diagnosis.

## Methods

The current study was undertaken and reported in accordance with the Guidelines for Reporting on Articles on Psychiatry and Heart rate variability (GRAPH) [28], which provides a standardized set of criteria for reporting HRV studies in the biobehavioral sciences [see Additional file 1].

### Participants

Ninety-one participants (mean age = 19.70, age range: 17–29) were recruited for the present study including 27 who met diagnostic criteria for a DSM-IV anxiety disorder, and 64 control participants that did not meet any DSM-IV diagnostic criteria. Participants were recruited from an undergraduate participant pool and received course credit for participation. In a typical sample of undergraduate students, the prevalence of anxiety with clinical severity is relatively low. Therefore, in an effort to recruit participants experiencing high levels of anxiety, participants were recruited based on responses to the Depression Anxiety Stress Scales – Short Form (DASS-21) [29], a screening measure. At the beginning of semester, a cohort of undergraduate psychology students completed a battery of measures including the DASS-21, allowing for targeted recruitment. In the present study, targeted participants included those who scored in the severe-to-extremely severe range on anxiety scale of the DASS-21. After providing written informed consent, all participants were administered the Anxiety Disorders Interview Schedule-IV for DSM-IV (ADIS-IV) [30] by one of two trained doctoral students (JAC or DSQ) to assess whether participants met DSM-IV criteria for an anxiety disorder. At the time of data collection, JAC had had extensive experience in administering the ADIS-IV through his studies as part of the doctorate of clinical psychology programme. DSQ was a PhD candidate in psychology and was provided with training and supervision in the administration of the ADIS-IV by JAC and MJAA, a clinical psychologist and senior lecturer in clinical psychology. All participants were provided with details of multiple mental health services they could access after testing, if required (e.g., if they reported significant distress). Control group participants did not meet criteria for any psychiatric disorder. All aspects of the study were approved by The University of Sydney’s Human Research Ethics Committee.

Exclusion criteria for the study included self-reported chronic physical illness (e.g., cardiac illness, cancer, epilepsy, and diabetes mellitus), psychotropic medication, pregnancy or lactation, psychosis spectrum disorder, traumatic brain injury, substance or alcohol dependence. Participants were instructed not to consume caffeine or nicotine on the day of their laboratory visit. Body mass index (BMI; assessed with a standard scale and tape measure) and an assessment of alcohol intake using the Alcohol Use Disorders Identification Test (AUDIT) [31] were calculated due to previously reported relationships with HRV [19, 32].

### Measures

*Anxiety Disorders Interview Schedule for DSM-IV (ADIS-IV)* [30]. The ADIS-IV is a semi-structured clinical interview based on DSM-IV-TR criteria, and is designed as a diagnostic tool for Axis-I disorders including anxiety and mood disorders. A clinical diagnosis was indicated by a clinical severity rating (CSR) of at least four on the clinical scale. The CSR is a rating made by the interviewing clinician (JAC or DSQ) on a 0 to 8 scale based on current symptom severity, distress, and interference (0 = none, 2 = subclinical, 4 = clinically significant, 6 = moderately severe, 8 = most severe).

*Penn State Worry Questionnaire (PSWQ)* [33]*.* The PSWQ is a 16-item self-report questionnaire designed to assess intensity and excessive worry. Items include “my worries overwhelm me” and “I worry all the time”, and are presented on a 5-point Likert-type scale. The PSWQ has been shown to have good internal consistency [34] and well-established validity [35]. The internal consistency in this study was found to be good (*α* = .80).

*Depression Anxiety Stress Scales – Short Form (DASS-21)* [29]. The DASS-21 is a self report scale assessing levels of depression, anxiety, and stress over the previous week, and consists of three scales: depression (DASS-D), anxiety (DASS-A), and stress (DASS-S). Items are rated on a 4-point Likert-type scale, with higher scores reflecting higher levels of depression, anxiety, or stress. The measure displays good-to-excellent reliability and validity [36]. Internal consistency of the three subscales was found to be good in the current study (*α*’s ranged from .88–.95).

*The State-Trait Anxiety Inventory (revised STAI-Y)* [37]. The STAI-state subscale (STAI-S) is a brief scale, consisting of 20 items coded on a 4-point Likert scale, designed to measure transient emotional reactions. The STAI-S good concurrent validity (*α* = .75–.85) and test-retest reliability in general: *α* = .73–.86 [37].

### Definition of groups

First, participants were grouped by diagnostic category according to whether diagnostic criteria were met for a primary diagnosis of an anxiety disorder as assessed by the ADIS-IV. While the analyses compared clinically anxious participants with non-anxious controls, associations with different dimensional indices of symptom severity, including depression, a symptom that is frequently comorbid with anxiety, were of primary interest [38]. Therefore, all participants, regardless of disorder diagnosis, were also divided into groups of high and low levels of symptom severity for worry, depression, anxiety, and stress, as defined by the PSWQ and the depression and anxiety subscales of the DASS-21 respectively. The cut-off for the high levels of worry symptoms from the PSWQ was 45 [39]. The anxiety and depression subscales of the DASS-21 were categorised into high symptom severity (severe to extremely severe; scores ≥ 21 on the depression subscale, scores ≥ 15 on the anxiety subscale, and scores ≥ 26 on the stress subscale) and low symptom severity (normal to moderate; scores ≤ 20 on the depression subscale, scores ≤ 14 on the anxiety subscale, and scores ≤ 25 on the stress subscale), using severity labels previously described based on normative data [29].

### Procedure

After informed consent was obtained, participants were asked to complete the state-trait anxiety inventory for a measure of state anxiety (STAI-S). Thereafter, a brief medical and psychological history was obtained by trained doctoral students. Arterial blood pressure was also recorded. Participants then underwent the structured diagnostic interview (ADIS-IV), which varied in duration between 45 minutes and 90 minutes depending on clinical severity of the participant, after which a battery of questionnaires was completed. Electrocardiogram (ECG) electrodes were then attached and data was recorded for six minutes while participants were relaxed and in a seated position. No instructions were given to alter breathing to avoid confounding associated with visceral-medullary feedback.

### Physiological data recording and processing

Ag/AgCl electrodes were attached in a modified Lead-II formation (right clavicle and left iliac crest) with a reference electrode on the left clavicle, and connected to an ECG, which sampled at 1000 Hz (PowerLab 8/30: ADInstruments, Sydney, AUS). ECG R-R series was obtained by the identification of the zero-points after a local maximum of the first derivative series via dedicated software (HRV Module, Labchart, ADI). All relevant segments were visually inspected and corrected for false or undetected R-waves, movement artifacts, and ectopic beats using piecewise cubic spline interpolation, with assessor blind to group status. Participants exhibiting significant deviation from sinus rhythm and electromyographic or movement errors (i.e., > 0.5 % of total beats) were excluded from the study. A frequency domain measure approximating the activity of respiratory sinus arrhythmia (high frequency, .15Hz–.40Hz; HF) was calculated by Fast Fourier Transform using Welch's Periodogram (window width 256s, 50 % overlap, resampled at 4 Hz). This measure of HRV was chosen as it best reflects parasympathetic modulation of the heart [40]. HRV metrics were calculated using Kubios (v2.0, Biosignal Analysis and Medical Imaging Group, University of Kuopio, Finland). HF-HRV violated Shapiro–Wilk's test for normality (all *p*s < .05), so raw scores were log transformed.

### Statistical analysis

Analyses were conducted using the “perfect *t*-test” script [41] and “stats” package in the R statistical environment (version 3.2.2). Welch’s *t*-tests and Pearson’s Chi-square test compared differences between groups on relevant demographic variables, including age and gender, to assess whether groups differed on common factors known to impact HRV. Welch’s *t*-tests were also used to compare HF HRV between groups. The common language effect size was computed, giving the probability that one random group observation is higher than another random observation from the other group [42]. Hedges’ *g* was also calculated as an effect size measure; this measure is better suited to studies with small sample sizes than the Cohen's *d* measure [43]. Bayes Factors (BF_10_) were calculated to quantify evidence for the alternative hypothesis (H1) relative to the null hypothesis (H0) [44] with a non-informative Jeffreys prior placed on the variance of the normal population and a Cauchy prior placed on the standardized effect size. The Bayes factor *r*-scale prior (not to be confused with Pearson’s *r* correlation coefficient) was set at 0.5, as a small effect size was anticipated. A BF_10_ value < 0.33 provides strong or ‘substantial’ evidence for the null hypothesis, over 3 provides strong evidence for the alternative hypothesis and between 0.33 and 3 provides only anecdotal support either way [45]. To investigate the relationship between HRV and variables of interest, correlational analyses were conducted across all participants with two-tailed Pearson correlations and Bayes Factors (putting a uniform prior on rho) using the JASP statistical package (version 0.7.5.5) [46]. The importance of reporting effect sizes regardless of statistical significance has been highlighted previously [5, 47, 48], and these recommendations are followed here. The correlation coefficient was interpreted as large when *r* = .5, medium when *r* = .3, and small when *r* = .10, while Hedges’ *g* was interpreted as large when *g* = .80, medium when *g* = .50, and small when *g* = .20.

## Results

### Participant characteristics

After blinded inspection of the ECG data, eight participants were excluded due to artefacts or significant deviations from sinus rhythm that comprised more than 0.5 % of total beats over the six-minute recording (ECG data available upon request), leaving a sample of 83 participants. Table 1 presents the participant characteristics for the control and clinical groups. There were no significant differences between groups on age, gender, and alcohol use. The clinical group had a significantly lower BMI than the control group (*p* = 0.04, Hedges’ *g* = 0.44; Table 1), however, a BF_10_ of 1.78 indicates the data are only 1.78 times more likely under the alternative hypothesis (than under the null hypothesis) providing only anecdotal evidence for the alternative hypothesis. Moreover, BMI was not significantly correlated with HRV (*r =* 0.02; 95 % CI: -.2 to .24; *p* = .84), with the BF providing substantial evidence that these variables are not related (BF_10_ = .14). These findings suggest that group differences in BMI are unlikely to contribute to differences in HRV in the present sample. As expected, clinical participants exhibited higher scores on all psychological measures of anxiety, depression, worry, and stress, relative to controls (all *p’*s < .001, see Table 1).

**Table 1:** Participant demographic and symptom characteristics

	Clinical (*n* = 25)	Control (*n* = 58)	*p*-values
Age in years^a^	19.71 (2.8)	19.56 (2.62)	.83
N of females (%)	76	58.6	.13
AUDIT	6.44 (5.87)	7.84 (5.92)	.32
Systolic BP^b^	119.32 (14.53)	122.19 (13.42)	.4
Diastolic BP^b^	79.76 (13.15)	75.81 (11.18)	.2
BMI^b^	21.31 (2.97)	23.01 (4.16)	.04
*Symptom Measures*			
PSWQ^c^	60.5 (11.77)	42 (11.85)	<.001
DASS D^d^	20.5 (12.75)	6.93 (8.41)	<.001
DASS A^d^	18.67 (10.74)	4.93 (6.23)	<.001
DASS S^d^	24.67 (10)	10.62 (8.16)	<.001
STAI S^e^	47.12 (11.37)	32.96 (7.74)	<.001

Note: Means and standard deviations (in parentheses) are presented for continuous data; *AUDIT* Alcohol Use Disorders Identification Test, *BMI* Body mass index, *PSQW* Penn State Worry Questionnaire, *DASS D* Depression, Anxiety and Stress Scale (short-form) depression subscale, *DASS21-A* Depression, Anxiety and Stress Scale (short-form) anxiety subscale, *DASS A* Depression, Anxiety and Stress Scale (short-form) stress subscale, *STAI S* State-Trait Anxiety Inventory – State scale. ^a^Clinical *n* = 24, Control *n* = 55; ^b^Clinical *n* = 25, Control n = 57; ^c^Clinical *n* = 24, Control *n* = 57; ^d^Clinical *n* = 24, Control *n* = 58; ^e^Clinical *n* = 25, Control *n* = 55

Overall, 25 participants met diagnostic criteria for a primary anxiety disorder, including PD (*n* = 3), GAD (*n* = 8), PTSD (*n* = 1), Social anxiety disorder (*n* = 12), and Obsessive compulsive disorder (*n* = 1). The mean CSR of these 25 participants was 4.92 (SD = 1.08) indicating clinical severity in this sample to be mild-to-moderate. While 17 of 25 clinical participants did not suffer from a comorbid anxiety disorder, six participants suffered from two anxiety disorders, and two participants suffered from three anxiety disorders. Division of groups were defined by symptom severity as follows: high depression (*n* = 17; including *n* = 12 from the clinical group) and low depression (*n* = 66; including *n* = 13 from the clinical group); high anxiety (*n* = 20; including *n* = 16 from the clinical group), low anxiety (*n* = 63; including *n* = 9 from the clinical group), and high stress (*n* = 15; including *n* = 12 from the clinical group) and low stress (*n* = 67; including *n* = 12 from the clinical group); and high worry (*n* = 41; including 22 from the clinical group) and low worry (*n* = 42; including *n* = 3 from the clinical group). These divisions were based on well-established cut-offs described earlier. Given the dimensional nature of anxiety and depression [38], and that some individuals report high levels of depression/anxiety without meeting diagnostic criteria, these groups were defined regardless of clinical status. Of note, some participants both met criteria for a primary anxiety disorder, and also fell in the low anxiety group, which may seem counterintuitive. These participants likely reflect those who met criteria for an anxiety disorder without symptoms of somatic anxiety as assessed by the anxiety subscale (e.g., GAD).

### Differences in HRV between groups

**Fig. 1:**
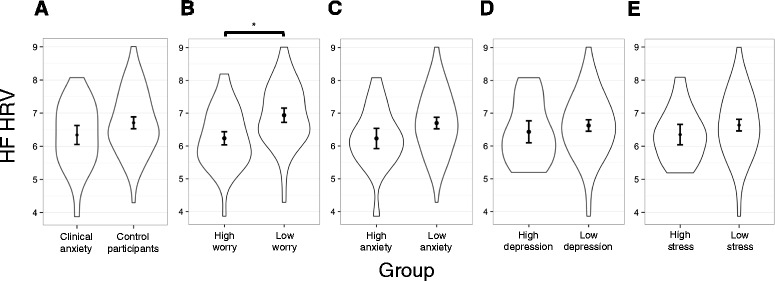
Violin plots with means and 95 % confidence intervals for HF-HRV. The following variables categories are shown: Clinically anxious vs. Control participants (**a**), Low vs. High worry (**b**), Low vs. High anxiety (**c**), Low vs. High depression (**d**), and Low vs. High stress (**e**). Violin plots illustrate the distribution of data by showing the probability density of the data at different values. HF-HRV = Absolute high frequency HRV. *p < 0.001

The clinical anxiety group displayed lower HF-HRV (*M* = 6.34, *SD* = .99, *n* = 25) than controls (*M* = 6.71, *SD* = .97, *n = 58*) at trend levels [*t*(44.89) = -1.57; *p* = .1; Hedges’ *g* = -.37; 95 % CI (-0.85, 0.1); Fig. 1a]. According to the common language effect size, the likelihood that the HF HRV of a random person in the clinical anxiety group is smaller than the HF HRV of a random person in the control group is 60 %. The BF_10_ was 0.84 indicating that the data are 0.84 times more likely under the alternative hypothesis, than under the null hypothesis, providing anecdotal evidence for the null hypothesis.

The high worry group displayed significantly reduced HF-HRV (*M* = 6.24, *SD* = .9, *n* = 41) relative to the low worry group (*M* = 6.94, *SD* = .96, *n* = 40; *t*(78.33) = -3.4, *p* = .001; Fig. 1b), a finding associated with a medium effect size [Hedges’ *g* = -.75, 95 % CI (-1.2, -0.3)]. According to the common language effect size, the likelihood that the HF HRV of a random person in the worry group is smaller than the HF HRV of a random person in the low worry group is 70 %. The BF_10_ of 28.16 further indicates that the data are 28.16 times more likely under the alternative hypothesis, than under the null hypothesis, providing strong evidence for alternative hypothesis.

The high anxiety group displayed reduced HF-HRV (*M* = 6.23, *SD* = 0.93, *n* = 20) relative to the low anxiety group (*M* = 6.7, *SD* = 0.98, *n* = 62), a finding that bordered the threshold for significance [*t*(33.32) = -1.94, *p* = .06, Hedges’ *g* = -.48; 95 % CI (-1.01, 0.01); Fig. 1c]. According to the common language effect size, the likelihood that the HF HRV of a random person in the high anxiety group is smaller than the HF HRV of a random person in the low anxiety group is 64 %. The BF_10_ was 1.42 indicating that the data are 1.42 times more likely under the alternative hypothesis, than under the null hypothesis, providing only anecdotal evidence for the alternative hypothesis.

There was no significant difference in HF-HRV between those categorised with high (*M* = 6.43, *SD* = 0.92, *n* = 17) and low (*M* = 6.63, *SD* = 1, *n* = 65) levels of depression severity [t(26.73) = -0.75, *p* = 0.46 Hedges’ *g* = -.19; 95 % CI (-.74, 0.33); Fig. 1d]. According to the common language effect size, the likelihood that HF-HRV of a person selected at random from the high depression group is smaller than one selected at random from the low depression group is 56 %. The BF_10_ was 0.44, indicating that the data are only 0.44 times more likely under the alternative hypothesis, than under the null hypothesis, providing only anecdotal evidence for the null hypothesis.

There was also no difference in HF-HRV between those categorised as high (*M* = 6.35, *SD* = 0.79, *n* = 15) and low (*M* = 6.64, *SD* = 1.02, *n* = 67) on stress [*t*(25.62) = -1.2, *p* = 0.240, Hedges' *g* = -0.29, 95 % CI (-0.91, 0.22); Fig. 1e]. According to the common language effect size, the likelihood that the HF-HRV of a random person in the high stress group is smaller than the HF HRV of a random person in the low stress group is 59 %. The BF_10_ was 0.62, which indicates the data are 0.62 times more likely under the alternative hypothesis, than under the null hypothesis, providing only anecdotal evidence for the null hypothesis.

### Associations between symptom severity measures and HRV

**Fig. 2:**
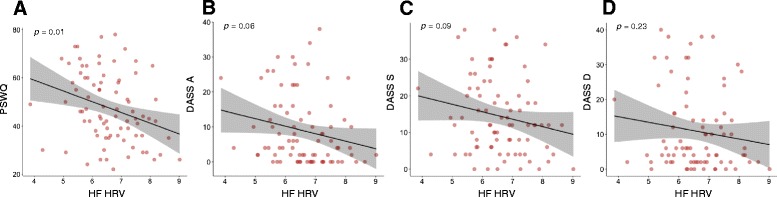
A scatterplot visualising the association between HRV and worry (**a**), anxiety (**b**), stress (**c**), and depression (**d**) symptom severity. A line of best fit with 95 % confidence region was overlaid on the scatterplots to illustrate data trends. DASS A = DASS anxiety score; DASS S = DASS stress score, DASS D = DASS depression score; PSWQ = Penn State worry questionnaire score

Bivariate correlations examined the relationship between symptom severity measures and HF-HRV at rest. The relationship between HF-HRV and worry (indexed by the PSWQ) was significantly inversely correlated (*r = -*0.31; 95 % CI: -.49 to -.10; *p* = .01; Fig. 2a), with the BF providing substantial evidence that these variables are inversely associated (BF_10_ = 5.9). Pearson’s correlation coefficient was indicative of a moderate effect size. There was no significant relationship between HF-HRV and anxiety (*r = -*0.21; 95 % CI: -.41 to -.01; *p* = .06; Fig. 2b) or stress (*r = -*0.19; 95 % CI: -.39 to .03; *p* = .09; Fig. 2c), with the BFs providing only anecdotal evidence that these variables are negatively associated (anxiety BF_10_ = .83; stress BF_10_ = .58). There was also no significant relationship between HF-HRV and depression (*r = -*0.14; 95 % CI: -.34 to .08; *p* = .23; Fig. 2d), and the BF provided substantial evidence that these variables are not related (BF_10_ = .28).

## Discussion

This study examined whether HRV in participants who fulfilled DSM-IV criteria for an anxiety disorder differed from control participants at resting state, and whether HRV reductions were more reliably associated with measures of depression, anxiety, stress, and worry, consistent with dimensional-trait models of psychopathology. HRV was reduced in clinically anxious participants relative to controls, although this difference only bordered on statistical significance. While prior research has demonstrated that HRV is reduced across anxiety disorders, there are variations across disorders in the degree to which this effect has been observed [25]. In fact, a recent study [26] across common mental disorders has demonstrated that only generalised anxiety disorder may display HRV reductions after many potential confounding factors are controlled. Heterogeneity across multiple anxiety disorders is one explanation for borderline findings between those with and without an anxiety disorder.

In the present study, all measures of symptom severity had an inverse relationship with HRV. However, only the PSWQ, an index of worry and cardinal feature of GAD, was observed to correlate significantly with HRV. These correlational analyses were complimented by between-group findings indicating that high worriers displayed significantly reduced HRV relative to low worriers, a finding associated with a large effect size. These findings were also complimented by strong and substantial evidence from Bayesian analyses, and provide convergent support for results from past studies, which have indicated that generalised anxiety disorder may be characterised by the most robust reductions in HRV [26, 49]. Our recent meta-analysis on the association between the anxiety disorders and HRV [25] also observed that HRV reductions in GAD were associated with a large effect size, while others have observed a significant inverse relationship between anxiety symptom severity in GAD patients and HRV [50]. Interestingly, recent evidence has also linked functional brain mechanisms associated with worry and rumination in GAD patients to reductions in HRV [51]. The source of worry in high worriers is not an external stressors, but cognitions about future threats [52]. Accordingly, pathological worry is distinguished by its chronicity, in contrast with more phasic forms of anxiety, such as panic [53]. Moreover, it has been suggested that anxiety in GAD is recognised as reflecting a long-term trait, or anxious temperament [54, 55]. It may be the chronic nature of worry symptomatology that leads to long-term withdrawal of the parasympathetic nervous system and persistent HRV reductions [26, 56]. The current finding of reduced HRV in high worriers is important considering the literature documenting the role of worry and GAD in cardiovascular risk [57].

### Study limitations

Some limitations of the present research should be noted. First, the specific impact of nosologically distinct anxiety disorders on HRV was not assessed. However, recent research on anxiety and HRV has also suggested that diminished HRV may represent a shared feature of anxiety disorders [50]. HRV may therefore provide a transdiagnostic psychophysiological marker of anxiety psychopathology. The investigation of common autonomic features across anxiety disorders – such as HRV – may also be a more ecologically valid method given the high level of co-morbidity across distinct disorders [58].

Second, while it is known that respiration parameters including rate and depth can affect HRV [59, 60], these factors were not accounted for throughout the experiment. While a commonly employed resolution to this problem has been to use paced breathing (e.g., [61]), the control of breathing may itself change HRV due to cortical involvement and adjusting visceral-medullary feedback [62]. Furthermore, some studies have reported that paced breathing may not provide any additional insights into autonomic function, over that provided when participants are spontaneously breathing [63–65].

## Conclusions

The present research indicates that although resting state HRV is reduced in individuals diagnosed with an anxiety disorder, the dimensional symptom of worry may actually be driving the observed HRV reductions, at least in the anxiety disorders. This finding provides support for several lines of research leading to proposals characterising vagal function – indexed by HF-HRV – as a physical pathway linking mental and physical health [13, 66]. Vagal function is considered to reflect a physical link because it not only appears to lay a physiological foundation from which psychological flexibility may arise, but it also has been shown to play an important regulatory role over a variety of physiological systems, including the sympathetic nervous system, the hypothalamic-pituitary-adrenal axis and inflammatory processes. Furthermore, our findings lend support to a recent suggestion [67] that HRV may be considered to index certain dimensional-traits underpinning psychiatric disorders that transcend diagnostic labels. In this regard, findings from the present study demonstrate that HRV indexes worry in individuals spanning the non-clinical to clinical spectrum. Future research should explore the impact of worry symptoms on HRV in other psychopathologies, such as major depressive disorder. The establishment of HRV as reliable transdiagnostic biomarker for worry may help facilitate the development of novel treatments (e.g., Non-invasive transcutaneous vagus nerve stimulation) and the identification of specific subgroups that are more likely to respond to such treatments. Finally, our results provide support for alternative frameworks for understanding psychiatric disorders, such as the Research Domain Criteria (or RDoC), and identify HRV as a particularly useful index of psychopathology that may index cognitive dysfunctions (i.e. excessive worry) leading to subsequent ‘wear and tear’ on the human body.

## Abbreviations

ADIS-IV, Anxiety Disorders Interview Schedule for DSM-IV; BF, Bayes factor; CSR, Clinical severity rating; CVD, Cardiovascular disease; OCD, Obsessive-compulsive disorder; DASS-21, Depression Anxiety Stress Scales – Short form; DASS-A, Depression Anxiety Stress Scales – Anxiety scale; DASS-D, Depression Anxiety Stress Scales – Depression scale; DASS-S, Depression Anxiety Stress Scales – Stress scale; GAD, Generalized anxiety disorder; HF, High frequency; HRV, Heart rate variability; PD, Panic disorder; PSWQ, Penn State Worry Questionnaire; RDoC, Research domain criteria; GRAPH, Guidelines for Reporting on Articles on Psychiatry and Heart rate variability; BMI, body mass index; AUDIT, Alcohol Use Disorders Identification Test; STAI-S, State-Trait Anxiety Inventory; ECG, electrocardiogram.

## Additional file

Additional file 1: Guidelines for Reporting on Articles on Psychiatry and Heart rate variability (GRAPH) checklist. A checklist of recommended items to report in a biobehavioral heart rate variability study. (PDF 64 kb)
